# The high frequency of esophageal disorders in Iranian patients with non-cardiac chest pain 

**Published:** 2018

**Authors:** Saeed Abdi, Roghayeh Sahraie, Habib Malekpour, Sara Ashatri, Somayeh Jahani-Sherafat, Majid Iranshahi, Mojgan Frootan

**Affiliations:** 1 *Gastroenterology and Liver Diseases Research Center, Research Institute for Gastroenterology and Liver Diseases, Shahid Beheshti University of Medical Sciences, Tehran, Iran*; 2 *Basic and Molecular Epidemiology of Gastrointestinal Disorders Research Center, Research Institute for Gastroenterology and Liver Diseases, Shahid Beheshti University of Medical Sciences, Tehran, Iran.*; 3 *Foodborne and Waterborne Diseases Research Center, Research Institute for Gastroenterology and Liver Diseases, Shahid Beheshti University of Medical Sciences, Tehran, Iran *; 4 *Imam Hossein Hospital, Shahid Beheshti University of Medical Sciences, Tehran, Iran*

**Keywords:** Non-cardiac heartburn, Gastro-esophageal reflux disease, Esophageal dysmotility, Functional chest pain

## Abstract

**Aim:**

The aim of this study was to evaluate the prevalence of gastrointestinal disorders in non-cardiac chest pain (NCCP) Iranian patients.

**Background:**

Gastro-esophageal reflux disease (GERD) is the most common cause of NCCP, which accounts for about one third of cases.

**Methods:**

This was a descriptive study on consecutive NCCP patients who referred to the gastroenterology clinic at the Taleghani Hospital, Tehran, Iran from 2015 to 2017. Medical history, physical examination and esophageal test including upper gastroenterology (UGI) endoscopy, esophageal manometry and 24 hour ambulatory esophageal pH monitoring were done for each participant.

**Results:**

The study included 102 patients, of which 58.9% were women, and the mean age of patients was 41.5 ± 11.2 years. The most common symptoms associated with chest pain were regurgitation in 28.4%, dysphagia in 23.5% and heartburn in 19.6% patients. UGI endoscopy was abnormal in 29.4% cases, esophageal manometry was abnormal in 61.7% cases and ambulatory pH monitoring was abnormal in 37.2% patients. Using UGI endoscopy and combined 24-h pH monitoring determined the prevalence of GERD 44.1% , and based on manometry the most frequent causes of NCCP was ineffective esophageal motility (IEM) in 19.6% patients with NCCP.

**Conclusion:**

Detecting etiology of NCCP allows healthcare providers to assure patients of the benign nature of their condition and provide appropriate treatment. It can also help prevent excessive hospital and physician visits as well as the costly and potentially risky testing which often results.

## Introduction

 Non-cardiac chest pain (NCCP) is a heterogeneous disorder associated with substantial health-care costs and resource utilization. NCCP is defined by recurrent episodes of chest pain that is indistinguishable from ischemic heart pain after a comprehensive evaluation and excluded coronary artery disease (1). There were very few population-based studies determining the prevalence of NCCP. These population based studies have reported that the prevalence of NCCP in western community ranges from 23% to 33% (2, 3). Little is known about these conditions in Asian countries. In a recently performed population survey in Hong Kong, using the Rose angina questionnaire, the prevalence of NCCP was found to be 13.9% in the general population (4). There seems to be no differences in the prevalence of NCCP between males and females. 

Identifying the cause of NCCP is still a problem in clinical practice, and because little is known regarding its pathophysiology, its mechanisms are numerous and overlapping. The main causes of NCCP are esophageal and psychiatric disorders, as well as musculoskeletal, pulmonary and miscellaneous alterations (5, 6). Gastro-esophageal reflux disease (GERD), esophageal motility disorders, esophageal visceral hypersensitivity, and psychological comorbidity are the main underlying mechanisms for NCCP (7, 8). Among them, GERD is the most common esophageal cause of NCCP (2, 9) with an estimated prevalence rate ranging from 40% to 60% in Western countries and 30% to 50% in Asia (10, 11). Esophageal motility disorders can be considered as an etiology of NCCP especially in non-GERD-related NCCP (12). Approximately 30% to 60% of patients with non-GERD-related NCCP are diagnosed as an esophageal motility disorder through esophageal manometric evaluation (13-15). Hypotensive lower esophageal sphincter (LES), diffuse esophageal spasm (DES), achalasia and Jackhammer as esophageal dysmotility disorders have been shown associated with chest pain (16). Esophageal visceral hypersensitivity is regarded as the presumed remaining etiology (17). Indeed, visceral hypersensitivity has been one of the most important pathophysiologic mechanisms in functional gastrointestinal disorders (16, 18, 19). Nevertheless, the etiology of NCCP still remains unclear in a significant portion of patients with NCCP.

Patients with NCCP have a poor quality of life and become frequent users of health-care resources. This can be an economic burden with respect to medical costs (20). Therefore, better knowledge of NCCP in the general population is necessary. The aim of this study was to examine the frequency of GERD and other esophageal disorders among NCCP patients. We evaluated the gastrointestinal disorders in NCCP patients by doing upper gastroenterology endoscopy and 24-h ambulatory pH monitoring to diagnose GERD and evaluating esophageal acid exposure. Furthermore, esophageal manometry in order to finding the presence of any esophageal dysmotility disorders was performed for all patients. 

## Methods

**Patients**

Consecutive patients with NCCP referred to the gastroenterology clinic at the Taleghani Hospital, Tehran, Iran from 2015 to 2017 enrolled in this study. NCCP was defined as recurrent chest pain that is not due to ischemic heart disease for more than three months duration, by the presence of at least two episodes of normal or non-obstructive coronary arteries; normal dobutamine stress echocardiography; or a negative exercise electrocardiogram and a cardiologist’s evaluation that the symptoms were not cardiac in origin (6). NCCP patients referred to the gastroenterology clinic for further investigations to understanding the mechanisms of NCCP. All patients filled out a detail questionnaire about demographic status such as; sex and age and symptoms including presence of typical symptoms of GERD (heartburn, regurgitation and dysphagia). Patients were excluded from study if they were under 18 year’s old, pregnant, using the proton-pump inhibitors (PPIs) seven days before the study, using H_2 _receptor antagonists 48 hours before the study, using non-steroid anti-inflammatory drugs (NASIDs) or aspirin, had a history of previous esophageal surgery and severe liver, lung renal or hematological diseases. Informed consent was obtained from all participants, and the study was approved by the Ethics Committee of the Research Institute for Gastroenterology and Liver Diseases, Shahid Beheshti University of Medical Sciences, Tehran, Iran.

**Study Design**

 All patients with NCCP underwent gastrointestinal investigations (upper GI endoscopy, esophageal manometry and 24-h esophageal pH monitoring) to understanding the mechanisms of NCCP.

**Upper Gastrointestinal Endoscopy**

 After an overnight fasting, upper gastrointestinal endoscopy was performed by two expert gastroenterologists who were blinded about the clinical status of the patients. All patients underwent endoscopy to diagnose reflux esophagitis and to exclude peptic ulcer disease by Olympus video-endoscope. The distal esophagus was carefully examined to detect the presence of lesions in the esophageal mucosa and its continuity was graded according to the Los Angeles Classification (21). 

The duodenal biopsies were interpreted by two expert pathologists who were not informed about the clinical status of the patients and interpreted small intestinal histological features, according to Marsh classification

**Esophageal Manometry**

Esophageal manometry was done according to the standardized protocol used in our center, using a water-perfused esophageal high-resolution manometry (HRM) (22, 23). HRM manometry was performed using a standard 3.5 mm diameter, water-perfused, 6-channel esophageal manometry catheter. Prior to the study, participants had fasted for 6 h. The HRM catheter passed Transnasally and positioned with the most distal channel in the stomach, the next most proximal in the lower esophageal sphincter (LES). Subsequently, participants were placed in a supine position and were asked to perform a series of 10 swallows of 5 mL of water. After the 10 swallows, participants were instructed not to swallow during 30 s, enabling a landmark recording to place the anatomical markers during analysis. The catheter was continuously perfused at 0.5 mL/min by a hydro-pneumatic infusion pump connected to the Solar UPS-2020 polygraph measurement system. Changes in intra esophageal pressure were converted into an electric signal by a transducer and then recorded on the computer (stationary motility systems, software version 8, GI Manometry). The manometric tracings were interpreted by a specialist and esophageal motility disorders were evaluated according to the Classification Criteria of Esophageal Motility Disorders (24, 25).

**24-h ambulatory pH monitoring**

PPI treatment was discontinued seven days before 24-h ambulatory pH monitoring. A mono-crystalline antimony pH catheter was passed to stomach via nasally, and the sensor positioned 5 cm above the lower esophageal sphincter (LES). The electrode catheter was connected to the portable unit and recording was started. Both electrodes were connected to a digital data recorder, which sampled pH activity at a rate of 4 Hz for at least 24 hours. Data were transferred from the recorder to a personal computer to analysis of pH data using Polygram NetTM Version: 4.01.525.45 software. The onset of a reflux episode was defined as a drop in the esophageal pH to less than 4 for at least four seconds, and its end as the first rise above 4. In case of a subsequent fall of pH below 4 within five seconds, both consecutive reflux episodes were interpreted as one single complex. The total percentage of time that the pH was below 4 (esophageal acid exposure time) was calculated for each patient GERD was considered to be present when the percentage exceeded 4.0%. Acid and non-acid reflux was diagnosed by pH-metry for each patient and GERD was divided into 3 subgroups; acid reflux (pH less than 4), weakly acidic reflux (pH between 4 and 7), and non-acid reflux/ alkaline reflux (pH upper than 7) (26).

**Statistical analysis**

Statistical analysis was performed using SPSS software (version 21.0, IBM Co., Chicago, IL). P-values <0.05 were considered as statistically significant. To explore the data, descriptive statistics; mean, standard deviation (SD), range, frequency, percentage (if continuous and normal distribution) and proportions (if categorical) were evaluated. 

## Results

**Patients and clinical data**

The study included 102 patients with NCCP, of which 58.9% were women, the mean age of patients was 41.5 ± 11.2 years. The most common symptoms associated to chest pain were, regurgitation in 29 (28.4%), dysphagia in 24 (23.5%) and heartburn in 20 (19.6%) the patients. the demographic and clinical data is provided in Table 1.

**Table 1:** Patients characteristics and clinical data of the study population (n=102)

Variables		
Sex	Male Female Total	45 (44.1)* 57 (55.9) 102 (100)
Age (years)	Male Female Total	40.5±11.4† 42.4±11.2 41.5±11.2
Symptoms	Heartburn Regurgitation Dysphagia	20 (19.6) 29 (28.4) 24 (23.5)

* Frequency (%);

† Mean ±standard deviation

**Upper gastrointestinal endoscopy findings**

Esophageal erosions during upper gastrointestinal endoscopy were presented in 30 (29.4%) of the patients with NCCP. GERD was presented in 14 (13.7%), inlet patch in 12 (11.8%), esophagus diverticulum in 3 (2.9%) patients and spastic lower esophageal sphincter (LES) was observed in only 1 (1%) patient. The remaining 72 (70.6%) patients had no significant endoscopic findings. In the group of patients with GERD, endoscopy revealed two grades of GERD that includes; GERD-A in 9 (64.3%) patients and GERD-B in 5 (35.7%) patients. Upper gastrointestinal endoscopy findings are provided in Table 2.

**Table 2:** Upper gastrointestinal endoscopy findings in study populations

Upper Endoscopy Findings	Frequency (%)
Normal	72 (70.6)
Gastro-esophageal reflux disease	14 (13.7)
Inlet Patch	12 (11.8)
Esophagus Diverticulum	3 (2.9%)
Spastic Lower Esophageal Sphincter	1 (1%)

**Table 3:** Esophageal manometry findings in study population

Esophageal Manometry Findings	Frequency (%)
Normal	39 (38.2)
Ineffective Esophageal Motility (IEM)*	20 (19.6)
Diffuse Esophageal Spasm (DES)	15 (14.7)
Hypotensive Lower Esophageal Sphincter (LES)	12 (11.8)
Achalasia	8 (7.8)
Esophageal Outflow Obstruction	5 (4.9)
Jackhammer Esophagus	3 (2.9)

* Non-specific esophageal motor disorders

**Esophageal manometry findings**

Esophageal manometry was done for all included patients, from 102 NCCP patients 63 (61.8%) patients had an abnormal esophageal manometry, with the following distribution; 20 (19.6%) of the patients had an ineffective esophageal motility (IEM), 15 (14.7%) diffuse/ distal esophageal spasms (DES), 12 (11.8%) a hypotensive lower esophageal sphincter (LES), 8 (7.8%) an achalasia, 5 (4.9%) an esophageal outflow obstruction and 3 (2.9%) of the patients had Jackhammer esophagus disorders. The remaining 39 (38.2%) patients had no significant manometry findings. Esophageal manometry findings are provided in Table 3.

**24-h ambulatory pH monitoring findings**

Abnormal pH monitoring were detected in 38 (37.2%) of the patients. Acid reflux and non-acid reflux detected in 31 (30.4%) and 7 (6.9%) patients, respectively. In the group of subjects with acid reflux, weakly acidic reflux was detected in 14 (45.1%) patients. The remaining 64 (62.7%) patients had no significant pH monitoring findings. 24-h ambulatory pH monitoring findings are provided in Table 4.

**Table 4:** 24-h ambulatory pH monitoring findings in study population

pH monitoring Findings	Frequency (%)
Normal	64 (62.7)
Acid reflux	31 (30.4)
Non-acid reflux	7 (6.9)

## Discussion

In this study, we examined the frequency of GERD and other esophageal disorders in the patients with NCCP, and to evaluate the mechanism of NCCP, patients underwent the upper gastrointestinal (UGI) endoscopy and 24-hour esophageal pH monitoring to diagnose GERD and evaluating esophageal acid exposure. In addition, esophageal manometry was used to determine esophageal motility disorders. The findings in our study indicated that the typical symptoms of reflux, heartburn, regurgitation and dysphasia were found in 19.6%, 28.4% and 23.5% of the patients, respectively. GERD during the UGI endoscopy and 24-h esophageal pH monitoring was determined in 14 (13.7%) and 38 (37.2%) patients, respectively. Moreover, 63 (61.8%) of patients were diagnosed with esophageal motility disorders based on esophageal manometry and the most frequent abnormal findings were non-specific esophageal motor disorders.

This discrepancy in findings of UGI endoscopy and 24-h esophageal pH monitoring is related to the two different methods and the pH monitoring has greater sensitivity and specificity than UGI endoscopy in the detection of GERD. Based on previous study all cases with reflux disorders; (GERD as reflux esophagitis and NERD as non-erosive reflux disorder) could not be diagnosed by upper endoscopy alone and required a 24-h esophageal pH monitoring and symptom questionnaire (27-29). Thus, ambulatory 24-h esophageal pH testing is particularly helpful in those patients who had normal endoscopy and to determine their acidity classifying them as acid or non-acid reflux (26). Using UGI endoscopy and combined 24-h esophageal pH monitoring, we found that the prevalence of GERD in patients with NCCP was 44.1%. This is in accordance with previous studies that reported the prevalence of GERD in NCCP patients about 40% to 48% in Asian countries (20, 30-32). However, studies from Western countries showed a higher prevalence of GERD in patients with NCCP up to 60% (6, 33). Ambulatory 24 hour esophageal pH testing studies have demonstrated that about half of NCCP patients have an abnormal esophageal acid exposure (7). Beedassy *et al*. evaluated 104 patients with NCCP and documented that 48% of them had an abnormal pH test (34). DeMeester *et al.* demonstrated that 46% of patients with chest pain had symptoms associated with an acid reflux event as documented during pH testing (35). Pandak *et al.* found an abnormal pH test in 42% of NCCP patients (36). In a study from China, 34.3% of the NCCP patients had at least 1 abnormal pH parameter. Similarly our results showed 37.2% patients with NCCP had an abnormal esophageal pH test.

Esophageal dysmotility disorders are important index for diagnosing NCPP and its relation and esophageal dysmotility is still a highly controversial topic. Some studies reported that in contrast to GERD a minority of patients with chest pain shows esophageal dysmotility disorders and demonstrated that approximately 30% of patients with NCCP had abnormal esophageal manometry (6, 7, 37, 38). In contrary, other studies reported the prevalence of esophageal motility disorders in NCCP patient’s upper than 60% (14, 15). The reason for the discrepancy between the results of these studies is unclear. Our findings showed 61.8% esophageal motility disorders in NCCP patients. This is in accordance with Doctoral Dissertation by Dr. Hilal in 2012, that reported the prevalence of esophageal motility disorders in NCCP patients 67% and diffuse esophageal spasm (DES) was the most prevalent abnormal finding in the NCCP group (15). Similar findings were also found by Lemme *et al*. reported that esophageal manometry showed abnormalities in 63% patients with NCCP, and the most frequent abnormal findings were non-specific esophageal motor disorders (39.7%), and hypotensive LES (35.7%) and then followed by nutcracker esophagus (10%), segmental spasm (7.3%), achalasia (4%), diffuse esophageal spasm (DES) (2.6%), and hypertensive LES (0.7%) (14). In contrast, Dekel *et al. *found the 30% of motility abnormality during esophageal manometry in patients with NCCP and identified hypotension of the lower esophageal sphincter (LES) as the most frequent esophageal dysmotility, followed by hypertension of the LES, non-specific motor disorders, and nutcracker esophagus (39). Katz *et al.* reported that 28% of NCCP patient’s had abnormal motility and the most frequent esophageal dysmotility was nutcracker esophagus, followed by non-specific motor disorders, diffuse esophageal spasm, hypotension of the LES, and achalasia (13). Our analysis shows, the most frequent abnormality during manometry was ineffective esophageal motility (IEM) (19.6%) and then followed by diffuse esophageal spasm (14.7%), hypotensive LES (11.8%), achalasia (7.8%), esophageal outflow obstruction (4.9%) and Jackhammer (2.9%).

Although the most common cause of NCCP is reported to be esophageal, in origin we found that 70.2%, 38.2% and 62.7% of the patients had no significant abnormality findings during UGI endoscopy, esophageal manometry and pH monitoring, respectively. In these patients, no cause has been determined for chest pain, which is neither cardiac nor esophageal in origin. On the other hand, psychological comorbidity has been shown to be common in NCCP and affects up to 75% of patients. According to previous studies the prevalence of panic disorders, anxiety and major depression was more than 50% in NCCP patients (4, 40, 41). Therefore, ruling out psychiatric disorders during the initial evaluation is recommended.

In conclusion, GERD is the most common esophageal cause for NCCP patients. It should be carefully evaluated. 24-h esophageal pH monitoring and symptom questionnaire is particularly helpful in those patients who had normal endoscopy and to determine their acidity classifying them as acid or non-acid reflux. In patients with non-GERD-related NCCP, the most frequent abnormality during manometry was ineffective esophageal motility (IEM) and then followed by diffuse esophageal spasm. In addition, ruling out psychiatric disorders during the initial evaluation is recommended. In general, detecting an etiology of NCCP allows healthcare providers to assure patients of the benign nature of their condition and provide appropriate treatment.

## Conflict of interests

The authors declare that they have no conflict of interest.
